# Bis(3-methyl­phenolato-κ*O*)(nitros­yl-κ*N*)[tris­(3,5-dimethyl­pyrazol-1-yl-κ*N* ^2^)hydridoborato]molybdenum(II)

**DOI:** 10.1107/S1600536810033763

**Published:** 2010-09-11

**Authors:** Wojciech Nitek, Piotr P. Romańczyk, Tomasz Lubera, Andrzej J. Włodarczyk

**Affiliations:** a Faculty of Chemistry, Jagiellonian University, ul. R. Ingardena 3, 30-060 Kraków, Poland; b Faculty of Chemical Engineering and Technology, Cracow University of Technology, ul. Warszawska 24, 31-155 Kraków, Poland

## Abstract

The title complex, [Mo(C_15_H_22_BN_6_)(C_7_H_7_O)_2_(NO)], contains an {MoNO}^4^ core stabilized by κ^3^­-hydrotris­(3,5-dimethyl­pyrazol-1-yl)borate, [Tp^Me2^]^−^, and two anionic *m*-cresolate ligands, leading to a distorted octa­hedral geometry for the Mo atom. The short Mo—O bond lengths [1.935 (2) and 1.971 (2) Å], as well as large Mo—O—C*sp* ^2^ angles [134.2 (2) and 143.54 (19)°], indicate *d*π_Mo_—*p*π_O_ inter­actions, which are clearly weaker when compared with {Mo(NO)(Tp^Me2^)} alkoxides. The nitrosyl system is virtually linear [179.3 (3)°] with Mo—N and N—O bond lengths of 1.760 (2) and 1.205 (3) Å, respectively. Intra- and inter­molecular C—H_(Ph or CH_3_)_⋯π_(Ph)_ inter­actions between adjacent phenyl rings are found in the crystal structure (*d*_H⋯Ph_ in the range 2.743–2.886 Å). One of the Ph rings shows disorder, *i.e.* swinging in the ring plane.

## Related literature

The importance of this class of Mo complexes comes from the fact that some {MoNO}^4^ alkoxides are efficient catalysts in the cathodic reduction of CHCl_3_. For the synthesis, spectroscopic characterization and electrochemical properties of [tris­(3,5-dimethyl­pyrazol-1-yl)borato]nitro­sylmolybdenum(II) bis-cresolates, see: Włodarczyk *et al.* (2008*a* ▶). For the spectroscopic characterization of the mono-cresolate analogue of the title compound, see: McCleverty *et al.* (1983 ▶). For related structurally characterized {Mo(NO)(Tp^Me2^)}-alkoxides, see: Romańczyk *et al.* (2007 ▶); Włodarczyk *et al.* (2008*c* ▶). For the electrocatalytic activity of bis-alkoxide Mo nitro­syls in the reduction of CHCl_3_, see: Włodarczyk *et al.* (2008*b* ▶).
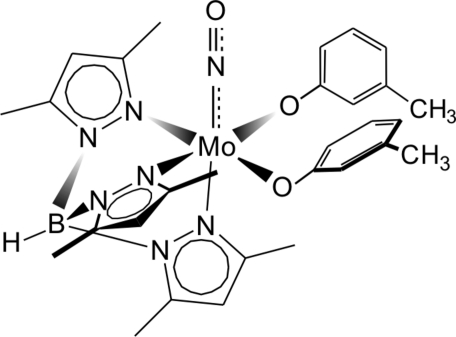


## Experimental

### 

#### Crystal data

[Mo(C_15_H_22_BN_6_)(C_7_H_7_O)_2_(NO)]
*M* *_r_* = 637.40
Triclinic, 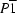

*a* = 8.041 (5) Å
*b* = 13.562 (5) Å
*c* = 14.591 (5) Å
α = 86.103 (5)°
β = 83.533 (5)°
γ = 74.597 (5)°
*V* = 1523.1 (12) Å^3^
*Z* = 2
Mo *K*α radiation
μ = 0.47 mm^−1^
*T* = 295 K
0.22 × 0.15 × 0.10 mm

#### Data collection

Nonius KappaCCD diffractometer
Absorption correction: multi-scan (*DENZO-SMN*; Otwinowski & Minor, 1997 ▶) *T*_min_ = 0.903, *T*_max_ = 0.954
12695 measured reflections
6901 independent reflections
5801 reflections with *I* > 2σ(*I*)
*R*_int_ = 0.024

#### Refinement

*R*[*F* ^2^ > 2σ(*F* ^2^)] = 0.041
*wR*(*F* ^2^) = 0.105
*S* = 1.08
6901 reflections
383 parameters
H atoms treated by a mixture of independent and constrained refinement
Δρ_max_ = 0.68 e Å^−3^
Δρ_min_ = −0.51 e Å^−3^

### 

Data collection: *COLLECT* (Nonius, 1998 ▶); cell refinement: *SCALEPACK* (Otwinowski & Minor, 1997 ▶); data reduction: *DENZO* (Otwinowski & Minor, 1997 ▶) and *SCALEPACK*; program(s) used to solve structure: *SIR97* (Altomare *et al.*, 1999 ▶); program(s) used to refine structure: *SHELXL97* (Sheldrick, 2008 ▶); molecular graphics: *ORTEP-3* (Farrugia, 1997 ▶); software used to prepare material for publication: *WinGX* (Farrugia, 1999 ▶).

## Supplementary Material

Crystal structure: contains datablocks global, I. DOI: 10.1107/S1600536810033763/rk2216sup1.cif

Structure factors: contains datablocks I. DOI: 10.1107/S1600536810033763/rk2216Isup2.hkl

Additional supplementary materials:  crystallographic information; 3D view; checkCIF report

## Figures and Tables

**Fig. 1.**
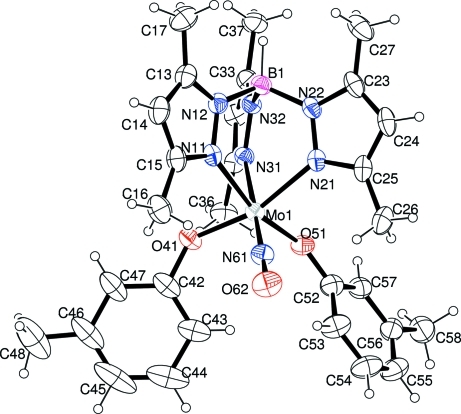
The molecular structure of title compound, showing the atom-numbering scheme. Displacement ellipsoids are drawn at the 30% probability level. H atoms are presented as a small spheres of arbitrary radius.

**Fig. 2.**
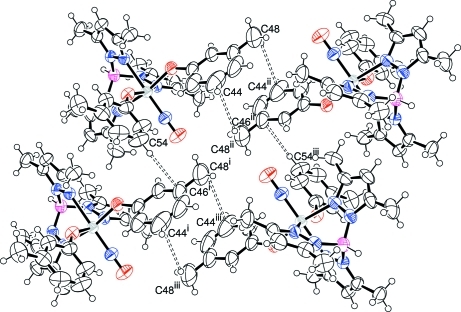
Intermolecular interactions in the crystal structure of the title compound.

## supplementary crystallographic information

### Comment

The stable 16*e* complexes containing {MoNO}^4^ core stabilized by tripodal hydrotris(3,5-dimethylpyrazol-1-yl)borate and two anionic co-ligands undergo easily reversible 1*e* reduction at a potential, *E*_1/2_, which can be tuned in the huge range of 2200 mV by selecting suitable co-ligands (Włodarczyk *et al.*, 2008*c*). The 17*e* species based on the {Mo(NO)(Tp^Me2^)(O-)_2_} moiety very efficiently catalyse the dehalogenation of CHCl_3_; their activity is strictly associated with the *E*_1/2_ value (Włodarczyk *et al.*, 2008*b*). The structural study of title complex is a part of a larger project concerning examination of molecules which may be potentially applied as electrocatalysts.

The title complex (Fig. 1) contains a *pseudo*-mirror plane of symmetry passing through Mo, NO, B and the N31/N32/C33/C34/C35 pyrazolyl ring (approximate *C_s_* symmetry which is reflected in ^1^H NMR spectrum). The longer Mo–O distances, *i.e.* weaker π-donation from O to Mo, in the bis-cresolato complex, than compared with those found for {Mo(NO)(Tp^Me2^)}-alkoxides, certainly result from additional electron delocalization to *sp*^2^-hybridized carbon (which is precluded in the case of the latter complexes, hence Mo–O_alkoxide_ av. distance is 1.88Å, see: Romańczyk *et al.*, 2007; Włodarczyk *et al.*, 2008*c*) and is reflected in hypsochromically shifted ν_NO_ band in the title complex (McCleverty *et al.*, 1983). The lengthening of the Mo1–N31 bond is attributed to the *trans*-influence of the NO group. Intermolecular Ph···Ph interactions between adjacent nearly perpendicular rings (C46B···H54^i^ distance is 2.873Å) and also between rings and methyl groups (C44···H48B^ii^ distance is 2.886Å), stabilize the crystal structure (Fig. 2), symmetry codes: (i) 1+*x*, *y*, *z*; (ii) 1-*x*, -*y*, -*z*. As mentioned above one of the Ph rings (linked with O41) is disordered, *i.e.* it swings in the ring plane.

### Experimental

The complex was synthesized following the literature from the reaction of [Mo(NO)(Tp^Me^)I_2_].C_6_H_5_CH_3_ and *m*-cresol in the presence of Et_3_N in boiling dichloromethane and characterized by mass spectrometry, IR, ^1^H NMR spectroscopy as well as cyclic voltammetry (Włodarczyk *et al.*, 2008*a*). A dark brown crystals were grown by slow evaporation of solvent from a dichloromethane/*n*-hexane solution.

### Refinement

The shape of the displacement elipsoids of atoms C43, C44, C45, C46, C47 and C48 suggests some kind of swinging disorder of the aromatic ring, however attempts to modelling of this disorder hasn't gave satisfactory results. All hydrogen atoms joined to carbon atoms of the discussed compound were positioned with an idealized geometry and refined using a riding model with C–H = 0.93Å and *U*_iso_(H) = 1.2*U*_eq_(C) for aromatic, C–H = 0.96Å and *U*_iso_(H) = 1.5*U*_eq_(C) for the methyl groups. Hydrogen atom joined to boron atom was found from the difference Fourier map and fully refined.

### Crystal data

**Table:**

[Mo(C_15_H_22_BN_6_)(C_7_H_7_O)_2_(NO)]	*Z* = 2
*M**_r_* = 637.40	*F*(000) = 660
Triclinic, *P*1	*D*_x_ = 1.390 Mg m^−^^3^
Hall symbol: -P 1	Mo *K*α radiation, λ = 0.71069 Å
*a* = 8.041 (5) Å	Cell parameters from 6763 reflections
*b* = 13.562 (5) Å	θ = 1.0–27.5°
*c* = 14.591 (5) Å	µ = 0.47 mm^−^^1^
α = 86.103 (5)°	*T* = 295 K
β = 83.533 (5)°	Prism, dark brown
γ = 74.597 (5)°	0.22 × 0.15 × 0.1 mm
*V* = 1523.1 (12) Å^3^	

### Data collection

**Table:**

Nonius KappaCCD diffractometer	5801 reflections with *I* > 2σ(*I*)
ω– and φ–scans	*R*_int_ = 0.024
Absorption correction: multi-scan (*DENZO-SMN*; Otwinowski & Minor, 1997)	θ_max_ = 27.4°, θ_min_ = 2.9°
*T*_min_ = 0.903, *T*_max_ = 0.954	*h* = −10→10
12695 measured reflections	*k* = −17→17
6901 independent reflections	*l* = −18→18

### Refinement

**Table:**

Refinement on *F*^2^	H atoms treated by a mixture of independent and constrained refinement
Least-squares matrix: full	*w* = 1/[σ^2^(*F*_o_^2^) + (0.0476*P*)^2^ + 0.7213*P*] where *P* = (*F*_o_^2^ + 2*F*_c_^2^)/3
*R*[*F*^2^ > 2σ(*F*^2^)] = 0.041	(Δ/σ)_max_ = 0.001
*wR*(*F*^2^) = 0.105	Δρ_max_ = 0.68 e Å^−^^3^
*S* = 1.08	Δρ_min_ = −0.51 e Å^−^^3^
6901 reflections	Extinction correction: *SHELXL97* (Sheldrick, 2008), Fc^*^=kFc[1+0.001xFc^2^λ^3^/sin(2θ)]^-1/4^
383 parameters	Extinction coefficient: 0.0117 (12)
0 restraints	

### Special details

**Table:**

Geometry. All s.u.'s (except the s.u. in the dihedral angle between two l.s. planes) are estimated using the full covariance matrix. The cell s.u.'s are taken into account individually in the estimation of s.u.'s in distances, angles and torsion angles; correlations between s.u.'s in cell parameters are only used when they are defined by crystal symmetry. An approximate (isotropic) treatment of cell s.u.'s is used for estimating s.u.'s involving l.s. planes.
Refinement. Refinement of *F*^2^ against ALL reflections. The weighted *R*-factor *wR* and goodness of fit *S* are based on *F*^2^, conventional *R*-factors *R* are based on *F*, with *F* set to zero for negative *F*^2^. The threshold expression of *F*^2^ > σ(*F*^2^) is used only for calculating *R*-factors(gt) *etc*. and is not relevant to the choice of reflections for refinement. *R*-factors based on *F*^2^ are statistically about twice as large as those based on *F*, and *R*-factors based on ALL data will be even larger.

### Fractional atomic coordinates and isotropic or equivalent isotropic displacement parameters (Å^2^)

**Table:**

	*x*	*y*	*z*	*U*_iso_*/*U*_eq_	
B1	0.0436 (4)	0.5567 (2)	0.2703 (2)	0.0491 (7)	
Mo1	0.07282 (3)	0.315037 (17)	0.229233 (15)	0.04338 (10)	
N11	0.1515 (3)	0.43407 (18)	0.13985 (15)	0.0483 (5)	
N12	0.1411 (3)	0.52830 (17)	0.17426 (15)	0.0478 (5)	
C13	0.2049 (4)	0.5870 (2)	0.1076 (2)	0.0584 (7)	
C14	0.2583 (4)	0.5300 (3)	0.0305 (2)	0.0667 (9)	
H14	0.3091	0.5504	−0.0255	0.08*	
C15	0.2223 (4)	0.4361 (3)	0.0517 (2)	0.0579 (7)	
C16	0.2551 (5)	0.3471 (3)	−0.0090 (2)	0.0787 (10)	
H16A	0.3245	0.2873	0.0206	0.118*	
H16B	0.3152	0.3617	−0.0669	0.118*	
H16C	0.1466	0.3351	−0.0197	0.118*	
C17	0.2090 (6)	0.6948 (3)	0.1220 (3)	0.0778 (10)	
H17A	0.0932	0.7386	0.1248	0.117*	
H17B	0.2791	0.7176	0.0717	0.117*	
H17C	0.2572	0.6972	0.1788	0.117*	
N21	−0.1565 (3)	0.44545 (18)	0.25060 (16)	0.0484 (5)	
N22	−0.1381 (3)	0.53995 (17)	0.26969 (16)	0.0491 (5)	
C23	−0.2964 (4)	0.6069 (2)	0.2796 (2)	0.0596 (8)	
C24	−0.4160 (4)	0.5555 (3)	0.2679 (2)	0.0655 (9)	
H24	−0.5355	0.5827	0.2715	0.079*	
C25	−0.3275 (4)	0.4552 (3)	0.2497 (2)	0.0566 (7)	
C26	−0.4001 (4)	0.3693 (3)	0.2310 (3)	0.0755 (10)	
H26A	−0.3376	0.3357	0.1769	0.113*	
H26B	−0.5202	0.3956	0.2212	0.113*	
H26C	−0.3894	0.3213	0.2829	0.113*	
C27	−0.3215 (5)	0.7166 (3)	0.2991 (3)	0.0823 (11)	
H27A	−0.268	0.7212	0.3536	0.123*	
H27B	−0.4432	0.7495	0.3083	0.123*	
H27C	−0.2694	0.7498	0.2478	0.123*	
N31	0.1762 (3)	0.38254 (16)	0.33866 (15)	0.0427 (5)	
N32	0.1396 (3)	0.48717 (16)	0.34540 (15)	0.0439 (5)	
C33	0.2048 (4)	0.5086 (2)	0.42083 (19)	0.0503 (6)	
C34	0.2859 (4)	0.4179 (2)	0.4624 (2)	0.0550 (7)	
H34	0.3435	0.4095	0.5154	0.066*	
C35	0.2656 (4)	0.3409 (2)	0.41023 (19)	0.0478 (6)	
C36	0.3312 (5)	0.2281 (2)	0.4255 (2)	0.0651 (8)	
H36A	0.3851	0.1979	0.3684	0.098*	
H36B	0.2362	0.1997	0.4479	0.098*	
H36C	0.4144	0.214	0.4701	0.098*	
C37	0.1823 (5)	0.6152 (3)	0.4507 (2)	0.0681 (9)	
H37A	0.2456	0.6505	0.4062	0.102*	
H37B	0.2254	0.6125	0.5098	0.102*	
H37C	0.0616	0.6508	0.4551	0.102*	
O41	0.3084 (3)	0.23057 (15)	0.21148 (14)	0.0557 (5)	
C42	0.3831 (5)	0.1373 (3)	0.1751 (2)	0.0686 (9)	
C43	0.3018 (7)	0.0590 (3)	0.1884 (3)	0.0873 (12)	
H43	0.1904	0.0701	0.2183	0.105*	
C44	0.3901 (10)	−0.0373 (4)	0.1560 (4)	0.123 (2)	
H44	0.3371	−0.0908	0.1644	0.148*	
C45	0.5542 (10)	−0.0536 (5)	0.1120 (4)	0.136 (3)	
H45	0.6119	−0.1184	0.0916	0.164*	
C46	0.6349 (7)	0.0241 (5)	0.0976 (3)	0.1104 (19)	
C47	0.5484 (5)	0.1210 (3)	0.1283 (3)	0.0871 (13)	
H47	0.6008	0.1747	0.1175	0.104*	
C48	0.8087 (8)	−0.0018 (6)	0.0543 (5)	0.169 (3)	
H48A	0.8837	0.0168	0.0928	0.254*	
H48B	0.8129	0.0344	−0.0044	0.254*	
H48C	0.846	−0.0742	0.0454	0.254*	
O51	−0.0196 (3)	0.24020 (14)	0.33482 (13)	0.0528 (5)	
C52	−0.1014 (4)	0.1689 (2)	0.3574 (2)	0.0585 (7)	
C53	−0.1504 (5)	0.1115 (3)	0.2944 (3)	0.0760 (10)	
H53	−0.1272	0.1236	0.2314	0.091*	
C54	−0.2322 (6)	0.0376 (3)	0.3251 (3)	0.0819 (11)	
H54	−0.2655	0.0004	0.2825	0.098*	
C55	−0.2668 (5)	0.0168 (3)	0.4192 (3)	0.0785 (11)	
H55	−0.3208	−0.0347	0.4388	0.094*	
C56	−0.2208 (4)	0.0727 (2)	0.4834 (3)	0.0641 (8)	
C57	−0.1373 (4)	0.1480 (2)	0.4516 (2)	0.0586 (7)	
H57	−0.1045	0.1855	0.4942	0.07*	
C58	−0.2607 (6)	0.0553 (3)	0.5851 (3)	0.0866 (12)	
H58A	−0.2717	−0.0133	0.5973	0.13*	
H58B	−0.3674	0.103	0.606	0.13*	
H58C	−0.1686	0.0649	0.6171	0.13*	
N61	−0.0075 (3)	0.26435 (19)	0.14084 (17)	0.0542 (6)	
O62	−0.0606 (4)	0.2291 (2)	0.07993 (17)	0.0799 (7)	
H1	0.035 (4)	0.641 (2)	0.283 (2)	0.053 (8)*	

### Atomic displacement parameters (Å^2^)

**Table:**

	*U*^11^	*U*^22^	*U*^33^	*U*^12^	*U*^13^	*U*^23^
B1	0.0534 (18)	0.0410 (15)	0.0496 (17)	−0.0084 (13)	−0.0011 (13)	−0.0013 (12)
Mo1	0.04415 (14)	0.04337 (14)	0.04167 (14)	−0.00827 (9)	−0.00681 (9)	−0.00342 (9)
N11	0.0433 (12)	0.0542 (13)	0.0430 (12)	−0.0067 (10)	−0.0002 (9)	−0.0021 (10)
N12	0.0471 (12)	0.0482 (12)	0.0464 (12)	−0.0117 (10)	−0.0030 (9)	0.0055 (9)
C13	0.0569 (17)	0.0637 (18)	0.0535 (17)	−0.0177 (14)	−0.0071 (13)	0.0157 (14)
C14	0.0624 (19)	0.087 (2)	0.0480 (16)	−0.0212 (17)	0.0012 (14)	0.0160 (16)
C15	0.0498 (16)	0.077 (2)	0.0420 (14)	−0.0106 (14)	−0.0006 (12)	0.0019 (13)
C16	0.084 (2)	0.096 (3)	0.0488 (18)	−0.013 (2)	0.0071 (16)	−0.0143 (17)
C17	0.094 (3)	0.070 (2)	0.075 (2)	−0.034 (2)	−0.017 (2)	0.0232 (18)
N21	0.0401 (11)	0.0553 (13)	0.0472 (12)	−0.0094 (10)	−0.0024 (9)	0.0010 (10)
N22	0.0443 (12)	0.0478 (12)	0.0483 (12)	−0.0014 (10)	−0.0022 (9)	0.0001 (10)
C23	0.0516 (16)	0.0640 (18)	0.0469 (15)	0.0106 (14)	−0.0009 (12)	0.0037 (13)
C24	0.0375 (14)	0.085 (2)	0.0600 (18)	0.0053 (15)	−0.0010 (13)	0.0065 (16)
C25	0.0395 (14)	0.078 (2)	0.0488 (15)	−0.0120 (14)	−0.0020 (11)	0.0077 (14)
C26	0.0498 (18)	0.094 (3)	0.087 (3)	−0.0272 (18)	−0.0093 (16)	0.010 (2)
C27	0.081 (3)	0.060 (2)	0.086 (3)	0.0149 (18)	−0.003 (2)	−0.0023 (18)
N31	0.0437 (11)	0.0393 (10)	0.0436 (11)	−0.0082 (9)	−0.0043 (9)	−0.0009 (9)
N32	0.0458 (12)	0.0412 (11)	0.0439 (11)	−0.0102 (9)	−0.0023 (9)	−0.0049 (9)
C33	0.0558 (16)	0.0546 (15)	0.0429 (14)	−0.0191 (13)	0.0003 (12)	−0.0080 (12)
C34	0.0639 (18)	0.0622 (17)	0.0422 (14)	−0.0191 (14)	−0.0125 (13)	−0.0028 (12)
C35	0.0481 (14)	0.0521 (15)	0.0432 (14)	−0.0125 (12)	−0.0076 (11)	0.0006 (11)
C36	0.079 (2)	0.0523 (16)	0.0625 (19)	−0.0095 (15)	−0.0258 (16)	0.0074 (14)
C37	0.082 (2)	0.0601 (18)	0.066 (2)	−0.0222 (17)	−0.0030 (17)	−0.0216 (15)
O41	0.0541 (11)	0.0497 (11)	0.0579 (12)	0.0004 (9)	−0.0110 (9)	−0.0110 (9)
C42	0.075 (2)	0.067 (2)	0.0519 (17)	0.0139 (17)	−0.0235 (15)	−0.0163 (15)
C43	0.125 (4)	0.0540 (19)	0.078 (3)	−0.009 (2)	−0.022 (2)	−0.0086 (18)
C44	0.195 (7)	0.059 (2)	0.111 (4)	−0.001 (3)	−0.059 (4)	−0.017 (2)
C45	0.175 (6)	0.094 (4)	0.108 (4)	0.057 (4)	−0.065 (4)	−0.056 (3)
C46	0.098 (3)	0.115 (4)	0.090 (3)	0.045 (3)	−0.036 (3)	−0.051 (3)
C47	0.070 (2)	0.097 (3)	0.078 (2)	0.021 (2)	−0.0231 (19)	−0.039 (2)
C48	0.122 (5)	0.201 (7)	0.151 (6)	0.043 (5)	−0.025 (4)	−0.094 (6)
O51	0.0647 (12)	0.0466 (10)	0.0489 (11)	−0.0155 (9)	−0.0124 (9)	−0.0007 (8)
C52	0.0586 (17)	0.0537 (16)	0.0634 (18)	−0.0119 (14)	−0.0161 (14)	0.0024 (14)
C53	0.100 (3)	0.073 (2)	0.068 (2)	−0.037 (2)	−0.030 (2)	0.0079 (17)
C54	0.105 (3)	0.069 (2)	0.088 (3)	−0.039 (2)	−0.042 (2)	0.0056 (19)
C55	0.081 (2)	0.060 (2)	0.103 (3)	−0.0299 (18)	−0.030 (2)	0.0211 (19)
C56	0.0578 (18)	0.0530 (17)	0.076 (2)	−0.0084 (14)	−0.0079 (15)	0.0113 (15)
C57	0.0628 (18)	0.0496 (16)	0.0618 (18)	−0.0116 (14)	−0.0101 (14)	0.0040 (13)
C58	0.090 (3)	0.073 (2)	0.087 (3)	−0.015 (2)	0.009 (2)	0.013 (2)
N61	0.0602 (15)	0.0551 (14)	0.0491 (13)	−0.0144 (11)	−0.0125 (11)	−0.0046 (11)
O62	0.102 (2)	0.0904 (18)	0.0586 (14)	−0.0349 (15)	−0.0271 (13)	−0.0076 (13)

### Geometric parameters (Å, °)

**Table:**

B1—N32	1.533 (4)	C33—C37	1.497 (4)
B1—N22	1.539 (4)	C34—C35	1.387 (4)
B1—N12	1.546 (4)	C34—H34	0.93
B1—H1	1.15 (3)	C35—C36	1.490 (4)
Mo1—N11	2.186 (2)	C36—H36A	0.96
Mo1—N21	2.200 (2)	C36—H36B	0.96
Mo1—N31	2.232 (2)	C36—H36C	0.96
Mo1—O41	1.935 (2)	C37—H37A	0.96
Mo1—O51	1.971 (2)	C37—H37B	0.96
Mo1—N61	1.760 (2)	C37—H37C	0.96
N11—C15	1.349 (4)	O41—C42	1.362 (4)
N11—N12	1.383 (3)	C42—C43	1.380 (6)
N12—C13	1.357 (4)	C42—C47	1.395 (6)
C13—C14	1.373 (5)	C43—C44	1.395 (6)
C13—C17	1.501 (5)	C43—H43	0.93
C14—C15	1.386 (5)	C44—C45	1.370 (10)
C14—H14	0.93	C44—H44	0.93
C15—C16	1.495 (5)	C45—C46	1.370 (9)
C16—H16A	0.96	C45—H45	0.93
C16—H16B	0.96	C46—C47	1.391 (6)
C16—H16C	0.96	C46—C48	1.430 (8)
C17—H17A	0.96	C47—H47	0.93
C17—H17B	0.96	C48—H48A	0.96
C17—H17C	0.96	C48—H48B	0.96
N21—C25	1.347 (4)	C48—H48C	0.96
N21—N22	1.379 (3)	O51—C52	1.311 (4)
N22—C23	1.352 (4)	C52—C53	1.396 (5)
C23—C24	1.360 (5)	C52—C57	1.397 (5)
C23—C27	1.490 (5)	C53—C54	1.364 (5)
C24—C25	1.385 (5)	C53—H53	0.93
C24—H24	0.93	C54—C55	1.394 (6)
C25—C26	1.488 (5)	C54—H54	0.93
C26—H26A	0.96	C55—C56	1.383 (5)
C26—H26B	0.96	C55—H55	0.93
C26—H26C	0.96	C56—C57	1.393 (4)
C27—H27A	0.96	C56—C58	1.499 (5)
C27—H27B	0.96	C57—H57	0.93
C27—H27C	0.96	C58—H58A	0.96
N31—C35	1.342 (3)	C58—H58B	0.96
N31—N32	1.378 (3)	C58—H58C	0.96
N32—C33	1.351 (3)	N61—O62	1.205 (3)
C33—C34	1.367 (4)		
			
N32—B1—N22	109.8 (2)	N32—N31—Mo1	120.68 (15)
N32—B1—N12	109.9 (2)	C33—N32—N31	109.4 (2)
N22—B1—N12	106.9 (2)	C33—N32—B1	131.7 (2)
N32—B1—H1	109.4 (15)	N31—N32—B1	118.9 (2)
N22—B1—H1	111.0 (15)	N32—C33—C34	107.9 (2)
N12—B1—H1	109.7 (15)	N32—C33—C37	123.3 (3)
N11—Mo1—N21	78.50 (9)	C34—C33—C37	128.7 (3)
N11—Mo1—N31	83.91 (8)	C33—C34—C35	106.6 (2)
N11—Mo1—O41	88.87 (9)	C33—C34—H34	126.7
N11—Mo1—O51	163.39 (8)	C35—C34—H34	126.7
N21—Mo1—N31	84.92 (9)	N31—C35—C34	109.6 (2)
N21—Mo1—O51	89.71 (9)	N31—C35—C36	122.3 (2)
N21—Mo1—O41	163.62 (9)	C34—C35—C36	128.1 (3)
N31—Mo1—N61	178.48 (10)	C35—C36—H36A	109.5
O41—Mo1—O51	100.29 (9)	C35—C36—H36B	109.5
N61—Mo1—O41	96.67 (11)	H36A—C36—H36B	109.5
N61—Mo1—O51	98.05 (10)	C35—C36—H36C	109.5
N61—Mo1—N11	94.57 (10)	H36A—C36—H36C	109.5
N61—Mo1—N21	94.71 (11)	H36B—C36—H36C	109.5
O41—Mo1—N31	83.40 (9)	C33—C37—H37A	109.5
O51—Mo1—N31	83.43 (8)	C33—C37—H37B	109.5
C15—N11—N12	106.6 (2)	H37A—C37—H37B	109.5
C15—N11—Mo1	133.1 (2)	C33—C37—H37C	109.5
N12—N11—Mo1	120.27 (16)	H37A—C37—H37C	109.5
C13—N12—N11	109.4 (2)	H37B—C37—H37C	109.5
C13—N12—B1	130.2 (3)	Mo1—O41—C42	134.2 (2)
N11—N12—B1	119.9 (2)	O41—C42—C43	121.2 (4)
N12—C13—C14	107.6 (3)	O41—C42—C47	118.3 (4)
N12—C13—C17	122.9 (3)	C43—C42—C47	120.5 (4)
C14—C13—C17	129.5 (3)	C42—C43—C44	118.7 (5)
C13—C14—C15	107.1 (3)	C42—C43—H43	120.6
C13—C14—H14	126.5	C44—C43—H43	120.6
C15—C14—H14	126.5	C45—C44—C43	120.6 (6)
N11—C15—C14	109.4 (3)	C45—C44—H44	119.7
N11—C15—C16	122.5 (3)	C43—C44—H44	119.7
C14—C15—C16	128.1 (3)	C44—C45—C46	121.1 (5)
C15—C16—H16A	109.5	C44—C45—H45	119.5
C15—C16—H16B	109.5	C46—C45—H45	119.5
H16A—C16—H16B	109.5	C45—C46—C47	119.4 (5)
C15—C16—H16C	109.5	C45—C46—C48	116.7 (6)
H16A—C16—H16C	109.5	C47—C46—C48	123.9 (7)
H16B—C16—H16C	109.5	C46—C47—C42	119.8 (5)
C13—C17—H17A	109.5	C46—C47—H47	120.1
C13—C17—H17B	109.5	C42—C47—H47	120.1
H17A—C17—H17B	109.5	C46—C48—H48A	109.5
C13—C17—H17C	109.5	C46—C48—H48B	109.5
H17A—C17—H17C	109.5	H48A—C48—H48B	109.5
H17B—C17—H17C	109.5	C46—C48—H48C	109.5
C25—N21—N22	107.0 (2)	H48A—C48—H48C	109.5
C25—N21—Mo1	132.7 (2)	H48B—C48—H48C	109.5
N22—N21—Mo1	120.31 (16)	Mo1—O51—C52	143.54 (19)
C23—N22—N21	109.1 (2)	O51—C52—C53	124.7 (3)
C23—N22—B1	130.5 (3)	O51—C52—C57	117.0 (3)
N21—N22—B1	120.2 (2)	C53—C52—C57	118.3 (3)
N22—C23—C24	107.8 (3)	C54—C53—C52	120.2 (4)
N22—C23—C27	122.6 (3)	C54—C53—H53	119.9
C24—C23—C27	129.6 (3)	C52—C53—H53	119.9
C23—C24—C25	107.5 (3)	C53—C54—C55	121.2 (3)
C23—C24—H24	126.2	C53—C54—H54	119.4
C25—C24—H24	126.2	C55—C54—H54	119.4
N21—C25—C24	108.6 (3)	C56—C55—C54	120.1 (3)
N21—C25—C26	123.3 (3)	C56—C55—H55	120
C24—C25—C26	128.1 (3)	C54—C55—H55	120
C25—C26—H26A	109.5	C55—C56—C57	118.4 (3)
C25—C26—H26B	109.5	C55—C56—C58	121.8 (3)
H26A—C26—H26B	109.5	C57—C56—C58	119.7 (3)
C25—C26—H26C	109.5	C56—C57—C52	121.8 (3)
H26A—C26—H26C	109.5	C56—C57—H57	119.1
H26B—C26—H26C	109.5	C52—C57—H57	119.1
C23—C27—H27A	109.5	C56—C58—H58A	109.5
C23—C27—H27B	109.5	C56—C58—H58B	109.5
H27A—C27—H27B	109.5	H58A—C58—H58B	109.5
C23—C27—H27C	109.5	C56—C58—H58C	109.5
H27A—C27—H27C	109.5	H58A—C58—H58C	109.5
H27B—C27—H27C	109.5	H58B—C58—H58C	109.5
C35—N31—N32	106.5 (2)	Mo1—N61—O62	179.3 (3)
C35—N31—Mo1	132.66 (18)		
			
N61—Mo1—N11—C15	−38.0 (3)	O51—Mo1—N31—C35	49.1 (2)
O41—Mo1—N11—C15	58.6 (3)	N11—Mo1—N31—C35	−141.7 (2)
O51—Mo1—N11—C15	−177.5 (3)	N21—Mo1—N31—C35	139.4 (2)
N21—Mo1—N11—C15	−131.9 (3)	O41—Mo1—N31—N32	133.34 (19)
N31—Mo1—N11—C15	142.0 (3)	O51—Mo1—N31—N32	−125.45 (19)
N61—Mo1—N11—N12	146.0 (2)	N11—Mo1—N31—N32	43.78 (18)
O41—Mo1—N11—N12	−117.38 (19)	N21—Mo1—N31—N32	−35.16 (18)
O51—Mo1—N11—N12	6.6 (4)	C35—N31—N32—C33	−0.6 (3)
N21—Mo1—N11—N12	52.13 (19)	Mo1—N31—N32—C33	175.22 (17)
N31—Mo1—N11—N12	−33.90 (19)	C35—N31—N32—B1	177.5 (2)
C15—N11—N12—C13	0.0 (3)	Mo1—N31—N32—B1	−6.7 (3)
Mo1—N11—N12—C13	176.92 (18)	N22—B1—N32—C33	−119.4 (3)
C15—N11—N12—B1	172.1 (2)	N12—B1—N32—C33	123.3 (3)
Mo1—N11—N12—B1	−11.0 (3)	N22—B1—N32—N31	63.0 (3)
N32—B1—N12—C13	−123.7 (3)	N12—B1—N32—N31	−54.3 (3)
N22—B1—N12—C13	117.2 (3)	N31—N32—C33—C34	0.8 (3)
N32—B1—N12—N11	66.1 (3)	B1—N32—C33—C34	−177.0 (3)
N22—B1—N12—N11	−53.0 (3)	N31—N32—C33—C37	−177.6 (3)
N11—N12—C13—C14	−0.7 (3)	B1—N32—C33—C37	4.6 (5)
B1—N12—C13—C14	−171.7 (3)	N32—C33—C34—C35	−0.7 (3)
N11—N12—C13—C17	178.7 (3)	C37—C33—C34—C35	177.6 (3)
B1—N12—C13—C17	7.7 (5)	N32—N31—C35—C34	0.1 (3)
N12—C13—C14—C15	1.0 (4)	Mo1—N31—C35—C34	−174.97 (19)
C17—C13—C14—C15	−178.3 (3)	N32—N31—C35—C36	−179.0 (3)
N12—N11—C15—C14	0.6 (3)	Mo1—N31—C35—C36	5.9 (4)
Mo1—N11—C15—C14	−175.7 (2)	C33—C34—C35—N31	0.4 (3)
N12—N11—C15—C16	179.9 (3)	C33—C34—C35—C36	179.4 (3)
Mo1—N11—C15—C16	3.6 (5)	N61—Mo1—O41—C42	−24.6 (3)
C13—C14—C15—N11	−1.0 (4)	O51—Mo1—O41—C42	74.9 (3)
C13—C14—C15—C16	179.8 (3)	N11—Mo1—O41—C42	−119.1 (3)
N61—Mo1—N21—C25	38.2 (3)	N21—Mo1—O41—C42	−158.3 (3)
O41—Mo1—N21—C25	172.2 (3)	N31—Mo1—O41—C42	156.9 (3)
O51—Mo1—N21—C25	−59.8 (3)	Mo1—O41—C42—C43	−37.3 (5)
N11—Mo1—N21—C25	132.0 (3)	Mo1—O41—C42—C47	146.1 (3)
N31—Mo1—N21—C25	−143.2 (3)	O41—C42—C43—C44	−174.9 (4)
N61—Mo1—N21—N22	−142.2 (2)	C47—C42—C43—C44	1.6 (6)
O41—Mo1—N21—N22	−8.3 (4)	C42—C43—C44—C45	0.0 (7)
O51—Mo1—N21—N22	119.75 (19)	C43—C44—C45—C46	−0.8 (8)
N11—Mo1—N21—N22	−48.47 (19)	C44—C45—C46—C47	0.0 (8)
N31—Mo1—N21—N22	36.33 (19)	C44—C45—C46—C48	177.6 (5)
C25—N21—N22—C23	−0.5 (3)	C45—C46—C47—C42	1.6 (6)
Mo1—N21—N22—C23	179.83 (18)	C48—C46—C47—C42	−175.8 (5)
C25—N21—N22—B1	−176.5 (2)	O41—C42—C47—C46	174.2 (3)
Mo1—N21—N22—B1	3.8 (3)	C43—C42—C47—C46	−2.4 (6)
N32—B1—N22—C23	122.9 (3)	N61—Mo1—O51—C52	5.9 (3)
N12—B1—N22—C23	−117.9 (3)	O41—Mo1—O51—C52	−92.5 (3)
N32—B1—N22—N21	−62.1 (3)	N11—Mo1—O51—C52	145.0 (3)
N12—B1—N22—N21	57.1 (3)	N21—Mo1—O51—C52	100.6 (3)
N21—N22—C23—C24	0.6 (3)	N31—Mo1—O51—C52	−174.5 (3)
B1—N22—C23—C24	176.1 (3)	Mo1—O51—C52—C53	2.9 (6)
N21—N22—C23—C27	−178.9 (3)	Mo1—O51—C52—C57	−178.7 (2)
B1—N22—C23—C27	−3.4 (5)	O51—C52—C53—C54	178.7 (4)
N22—C23—C24—C25	−0.5 (4)	C57—C52—C53—C54	0.4 (6)
C27—C23—C24—C25	179.0 (3)	C52—C53—C54—C55	−0.8 (6)
N22—N21—C25—C24	0.2 (3)	C53—C54—C55—C56	1.2 (6)
Mo1—N21—C25—C24	179.8 (2)	C54—C55—C56—C57	−1.2 (5)
N22—N21—C25—C26	179.7 (3)	C54—C55—C56—C58	178.0 (4)
Mo1—N21—C25—C26	−0.7 (4)	C55—C56—C57—C52	0.8 (5)
C23—C24—C25—N21	0.2 (4)	C58—C56—C57—C52	−178.3 (3)
C23—C24—C25—C26	−179.3 (3)	O51—C52—C57—C56	−178.9 (3)
O41—Mo1—N31—C35	−52.1 (2)	C53—C52—C57—C56	−0.4 (5)
